# Supplementary figures and images for: Sh3bp2 Gain-Of-Function Mutation Ameliorates Lupus Phenotypes in B6.MRL-Faslpr Mice
Source: Cells. 2019 Apr 30;8(5):402. doi: 10.3390/cells8050402 (PMC6562867; doi:10.3390/cells8050402)

## Slide 1
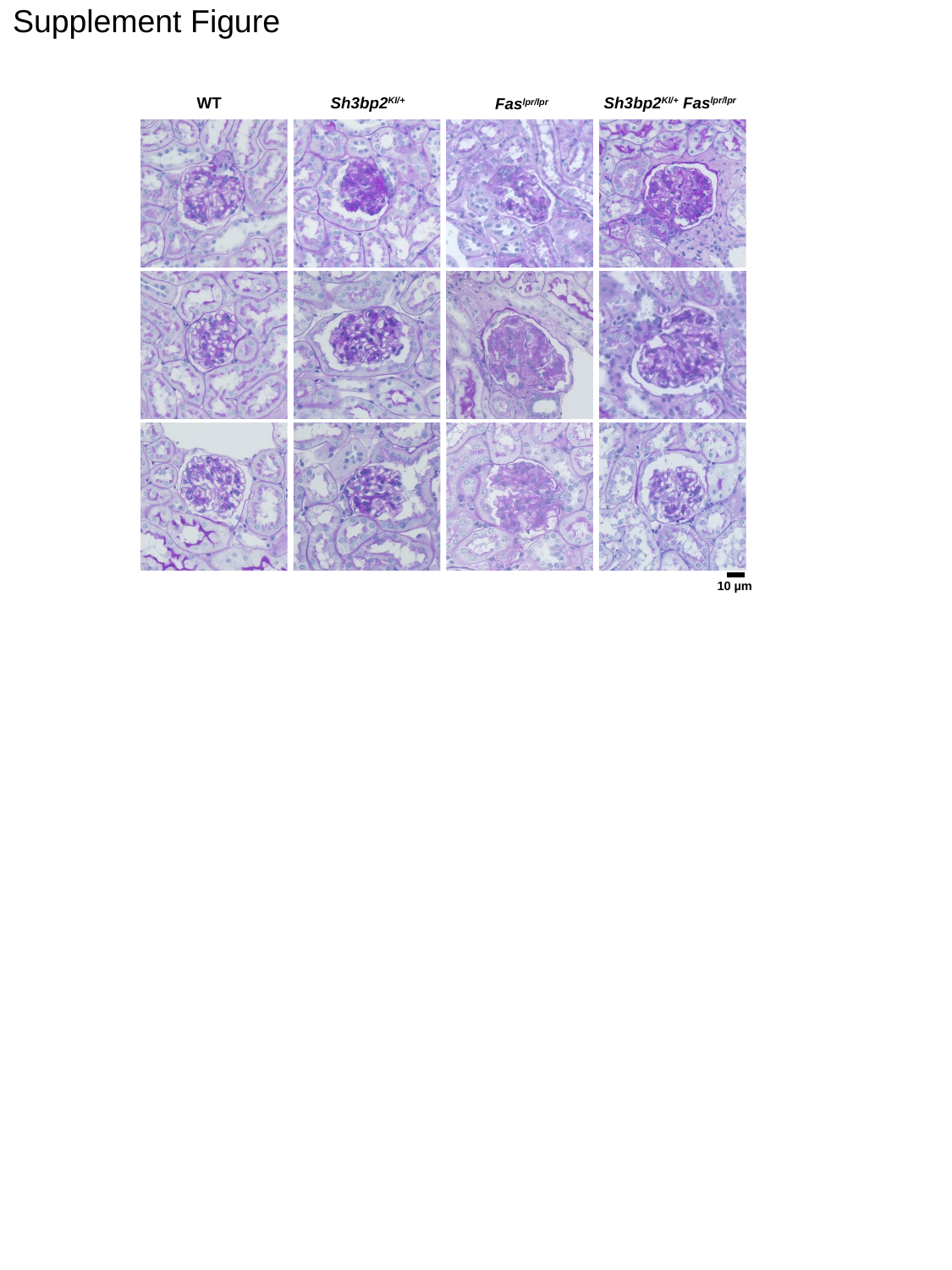

Supplement Figure
WT
Sh3bp2KI/+
Sh3bp2KI/+ Faslpr/lpr
Faslpr/lpr
10 µm

Supplement: Supplementary file 1 [file cells-08-00402-s001.zip › cells-479458-supplementary.pptx]
